# Mental health in Austrian psychotherapists during the COVID-19 pandemic

**DOI:** 10.3389/fpubh.2022.1011539

**Published:** 2022-11-08

**Authors:** Yvonne Schaffler, Stefan Kaltschik, Thomas Probst, Andrea Jesser, Christoph Pieh, Elke Humer

**Affiliations:** Department for Psychosomatic Medicine and Psychotherapy, University for Continuing Education Krems, Krems, Austria

**Keywords:** COVID-19, psychotherapists, wellbeing, depression, anxiety, insomnia, stress

## Abstract

Although the impact of the COVID-19 pandemic on mental health has been reported in different communities, little is known about the mental health of psychotherapists during the COVID-19 pandemic. This study aimed to assess mental health during the COVID-19 pandemic in Austrian psychotherapists and compare it with the general population. A total of *n* = 513 psychotherapists (80.5% women; mean age: 53.06 ± 9.94 years) took part in an online survey conducted from April to June 2022. At the same time, a representative sample (*N* = 1,031) of the Austrian general population was surveyed online. Indicators of mental health were mental wellbeing (WHO-5), depression (PHQ-2), anxiety (GAD-2), insomnia (ISI-2), and stress (PSS-10). The general population sample was matched according to age and gender with the psychotherapist's data using propensity scores, yielding a final sample of *n* = 513 (80.5% women; mean age: 52.33 ± 13.39 years). Psychotherapists showed lower odds for exceeding cut-offs for clinically relevant depressive, anxiety, insomnia and stress symptoms (0.34–0.58) compared to the general population. Further studies should elucidate the protective factors underlying these findings.

## Introduction

The outbreak of the coronavirus disease 2019 (COVID-19) pandemic in early 2020 became not only a global threat to physical health but also negatively affected people's mental wellbeing in different communities (1). Studies reported 2–8 times increased rates of depression, anxiety, insomnia, and stress among the general population since the COVID-19 pandemic (1–4). Healthcare workers seem even more burdened than the general population (5, 6), being not only vulnerable to experiencing physical exhaustion due to the extremely high workloads (7). They are at a higher risk of adverse mental health outcomes (6, 8), showing lower wellbeing and higher prevalences of depression (31 vs. 25%) and anxiety (31 vs. 27%) than the general population (4).

Previous studies evaluating mental health in health care professionals focused mainly on doctors and nurses (8–11), while the research on mental health in those professions providing mental healthcare is minimal. The few studies investigating mental health in mental healthcare professionals mainly focused on psychiatrists (12–14). These studies indicated that psychiatrists are exposed to several stressors, which might increase their risk for deprivation of mental health (12, 13). A study conducted in Germany during the first wave of the COVID-19 pandemic in hospital personnel also compared the moral distress of different professions (i.e., physicians, nurses, medical technical assistants, psychologists/psychotherapists, pastoral counselors). Moral distress was higher for nurses and medical technical assistants than physicians, psychologists/psychotherapists and pastoral counselors. However, no differences between physicians, psychologists/psychotherapists and pastoral counselors were observed (15).

Not only during the pandemic, then rather in general, mental health in psychotherapists remains largely understudied. The research conducted so far focused mainly on burnout and wellbeing (16), while only little is known about the prevalence of mental health disorders in psychotherapists. Mental health disorders are not only of greater interest from a clinical perspective, but also adopt more advanced operationalizations and assessments. The largest investigation conducted on mental health problems among mental health care professionals prior to the pandemic found an even higher lifetime prevalance (63%) of mental health symptoms compared to the estimates in the general population (41%) (17). High prevalences of mental disorders in mental healthcare providers are not only detrimental to the directly affected individual, but also negatively impact the entire psychotherapeutic process and thus patient care (18).

Scientific discourse on the mental health needs of psychotherapists themselves during the ongoing COVID-19 pandemic is rare. Next to the general societal impact, psychotherapists face additional unprecedented professional challenges. Among these challenges are implementing new treatment formats (telephone, internet), a higher likelihood of vicarious traumatisation and professional self-doubt (19). One of the few studies on mental health in psychotherapists evaluated stress levels and job anxiety in Austrian psychotherapists in the early weeks of the first COVID-19-associated lockdown. Results showed that previous experiences with teletherapy, the therapeutic format used during the lockdown, and changes in patient numbers compared to the times before the pandemic were not associated with job anxiety and perceived stress levels (20). Another study conducted during the early phase of the pandemic in the United States explored the extent to which the levels of perceived stress predict burnout in professional counselors (21). A strong positive association between stress levels and burnout was observed, emphasizing the importance for mental health care professionals to engage in self-care practices during the pandemic to mitigate the detrimental effects of stress. However, the abovementioned studies did not evaluate mental wellbeing, depression, anxiety and insomnia indicators.

In Austria, the general population's mental health was evaluated several times throughout the last two and a half years. A study conducted during the first COVID-19 lockdown (April 2020) revealed a substantial increase in mental health symptoms compared to data collected before the pandemic (22). The easing of protective measures was not followed by improved mental health 6 weeks (23) and 6 months (24) after the end of the first lockdown. During the second wave of the pandemic in Austria, further strict lockdown measures were in place, accompanied by a further increase in the prevalence of mental health disorders around the turn of the year 2020/2021 (25). Further lockdown measures accompanied Austria's third (the Beta variant) and fourth (the Delta variant) waves of infections. The fifth wave of infections (the Omicron variant) emerged end of December 2021 (26). However, although Austria—like most countries around the globe—lifted most protective measures in the spring of 2022, the latest data on mental health in the general population do not hint that the relaxation of measures is associated with improvements in mental health (27). Data collected end of April 2022 even observed higher rates of depression than in April 2020 and no improvement in anxiety, insomnia and stress symptoms (28).

With this surge in mental illnesses in the general population and associated increased demands for professional services (29), the role of psychotherapists becomes crucial in providing professional mental health care to people suffering from mental health problems.

Psychotherapists are not only confronted with an increasing number of patients seeking therapeutic support (30) but are also dealing with diverse and significant issues, as their patients present themselves with greater burdens and exacerbated mental health distress (21). Frequently addressed concerns are related to restrictions, fear of illness, unemployment, economic recession and worrying sociopolitical developments (31). It has been suggested that these aspects augment the risk of burnout amongst mental health practitioners (32).

However, whether psychotherapists face similar psychological challenges as the general population and whether these unprecedented challenges negatively impact the mental health of psychotherapists has not been assessed so far.

Therefore, our study aimed to examine mental wellbeing, perceived stress, depressive, anxiety and insomnia symptoms in Austrian psychotherapists during the COVID-19 pandemic and compare these mental health indicators in psychotherapists with the Austrian general population.

## Methods

### Design

An online survey among licensed Austrian psychotherapists was conducted between April 11 and May 31, 2022. The link to the survey was sent *via* e-mail to psychotherapists registered in the list of the Austrian Federal Ministry of Social Affairs, Health, Care and Consumer Protection (>11,000 psychotherapists registered in April 2022), providing a valid e-mail address (≈7,000 psychotherapists). Also, the Austrian Federal Association for Psychotherapy (ÖBVP) invited their members to participate in the survey. Psychotherapists' participation was voluntary, without incentives.

Between April 19 and 26, 2022, a representative sample of the Austrian general population was recruited from a pre-existing online access panel provided by Marketagent.com online research GmbH (Baden, Austria; certified under ISO 20252). Participants had to reside in Austria, and have access to the internet to participate in the study. Marketagent has about 130,000 registered panelists in Austria (33). Using quota sampling, *N* = 1,031 respondents were selected and invited based on quotas for the following key demographics: age, gender, age x gender, region, and educational level. As licensed psychotherapists need to be at least 28 years old in Austria (34), and the gender distribution is unequal [almost three-quarters are female (26)], all participants younger than 28 years of the general population were excluded. From the remaining sample, a subsample was matched with the psychotherapists‘ data *via* propensity score adjusting for age and gender. Accounting for confounding by the covariates age and gender by matching both datasets was also based on previous findings, showing higher mental health burden in women vs. men as well as younger vs. older individuals in the Austrian general population during the COVID-19 pandemic (22, 25).

This study was conducted following the Declaration of Helsinki and approved by the Ethics Committee of the University for Continuing Education Krems, Austria (Ethical numbers: EK GZ 26/2018-2021, EK GZ 11/2021-2024). All participants gave electronic informed consent to participate and complete the questionnaires.

### Measures

#### Sociodemographic variables

All participants were asked about gender, age and federal state. Psychotherapists were further asked about their years in the profession and the psychotherapy method they practice. In Austria, there are 23 methods accredited, which can be classified into psychodynamic, humanistic, systemic and behavioral orientations (35).

#### Wellbeing (WHO-5)

Wellbeing was assessed with the 5-item World Health Organization Wellbeing Index (WHO-5) (36). The WHO-5 comprises five positively phrased questions on wellbeing over the last 2 weeks that are self-rated on a six-point Likert scale from 0 (none of the time) to 5 (all of the time). The raw total score ranges from 0 to 25, with higher scores indicating higher wellbeing. As previously recommended (37), the scores were multiplied by 4 to translate into a percentage scale from 0 (absence of wellbeing) to 100 (maximal wellbeing). Cronbach's alpha was α = 0.85 in the psychotherapist's sample and α = 0.93 in the general population's age- and gender-matched subsample.

#### Perceived stress (PSS-10)

Perceived stress levels were measured with the Perceived Stress Scale (PSS-10). The 10 items of the PSS-10 measure stress on a five-point scale from 0 to 4, with a cut-off score of 14 defining moderate stress levels (38). Cronbach's alpha was α = 0.86 in the present psychotherapists' sample and α = 0.87 in the general population subsample.

#### Depressive symptoms (PHQ-2)

The first two items of the depression module of the Patient Health Questionnaire (39) were used to assess depressive symptoms over the past 2 weeks. The two self-rating items of the PHQ-2 ask about losing interest and pleasure and feeling down, depressed or hopeless. Response options range from “not at all” to “nearly every day” over the last 2 weeks, scored from 0 to 3, yielding a total score from 0 to 6, with higher values indicating more severe depressive symptoms. A cut-off point of at least 3 points is defined as clinically relevant depressive symptoms (40). Cronbach's alpha was α = 0.64 in the present psychotherapists' sample and α = 0.81 in the general population subsample.

#### Anxiety (GAD-2)

Anxiety symptoms were assessed with the two core items of the Generalized Anxiety Disorder scale (41, 42). The two self-rating items measure feelings of nervousness, anxiety or being on edge, and inability to stop or control worrying over the last 2 weeks on a four-point scale from 0 to 3. A cut-off score of 3 has been recommended to define clinically relevant anxiety symptoms (42). Cronbach's alpha was α = 0.68 in the present psychotherapists' sample and α = 0.84 in the general population subsample.

#### Insomnia (ISI-2)

Sleep quality was measured with a brief scale from the Insomnia Severity Index (ISI) (43). The two items of the ISI-2 measure satisfaction/dissatisfaction with current sleep patterns and interferences with daily functioning over the past 2 weeks on a five-point scale from 0 to 4, with a cut-off score of 6 pointing to insomnia disorder (44). Cronbach's alpha was α = 0.69 in the present psychotherapists' sample and α = 0.78 in the matched subsample of the general population.

### Sample size

Power analysis was performed with G^*^Power 3.1.9.4 (45). With an error type 1 of 0.05 and a power of 0.8 and an expected effect size of d = 0.4, a sample of 100 persons is required when the use of *T*-tests (two samples). However, as this study is part of a larger longitudinal study, including a second measurement point in spring 2023, we aimed to recruit a larger sample size in 2022, to enable repeated measures analysis with the psychotherapists participating at both time points. Assuming a response rate of 20% in 2023, the aim was to recruit at least 500 psychotherapists in 2022. From previous studies, we have seen numbers ranging from 200 to 1,500 psychotherapists participating in online surveys when all licensed Austrian psychotherapists were invited (30, 46). Therefore, the total population of licensed Austrian psychotherapists was invited to participate. This also has the benefit of maximizing external validity as much as possible.

### Statistical analyses

Descriptive statistics were conducted to describe the sociodemographic characteristics. Chi-squared tests and *t*-tests for independent samples were applied to assess differences in sociodemographic and professional characteristics between participating psychotherapists and the total population of licensed Austrian psychotherapists.

To account for confounding by covariates age and gender between the general population sample and the psychotherapists' sample propensity score matching was performed using the MatchIt package in R (47). Optimal pair matching without replacement was applied, and the propensity score was estimated with logistic regression. After matching all standardized mean differences for the covariates were below 0.01, standardized mean differences of gender were reduced from 0.74 to 0 and for age from 0.20 to 0.07. The sample size before matching was 1,352 (general population: 839, psychotherapists 513). The 326 units from the general population sample were discarded by matching, resulting in a sample with 513 units per comparison group.

Univariate analyses were applied using *T*-tests for independent samples and Chi-squared tests. *T*-tests were conducted to assess differences in mean values of mental health indicators between psychotherapists and the general population.

Chi-squared tests were conducted to analyse differences in the prevalence of clinically relevant depression, anxiety, insomnia and stress between psychotherapists and the general population.

Multivariable binary logistic regression was applied to account for the potential confounders age and gender. The mental health variables were the dependent variables and the group (psychotherapists vs. general population), gender (female vs. male) and age the predictors. Adjusted odds ratios (OR) and their 95% confidence intervals (CIs) were estimated to assess the statistical uncertainty.

Chi-squared tests, *T*-tests and logistic regression analyses were performed using SPSS version 26 (IBM Corp, Armonk, NY, USA). *P*-values of < 0.05 were considered statistically significant (2-sided tests). Cohen's d and the 95% CI were calculated as an effect size measure.

## Results

### Study sample characteristics

In total, *n* = 530 psychotherapists started the survey (response rate ≈ 7.6%) of whom *n* = 513 provided information on all outcome variables (completion rate *n* = 96.8%). Only psychotherapists providing information on all outcome variables were included in the analyses. Sociodemographic and professional characteristics are summarized in Table 1. They were 53.06 ± 9.94 years old, and 80.5% were female. Compared to the list of all licensed Austrian psychotherapists, more female psychotherapists (80.5 vs. 73.8%; *P* = 0.001) and those with less professional experience (12.40 vs. 16.24 years; *P* < 0.001) participated in the survey. Regarding theoretical orientation, psychodynamic and behavioral psychotherapists were underrepresented, whereas humanistic psychotherapists were overrepresented (*P* < 0.001). Also, participation was disproportionately higher among Lower Austrian psychotherapists (18.9% in the survey vs. 12.8% in the total sample; *P* < 0.001).

**Table 1:** Study sample characteristics (*n* = 513).

	**Participating** **psychothera** **pists** **(*n* = 513)**	**Licensed psychothe** **rapists** **(*n* = 11,156)**	**Statistics**
**Gender**			
Female, % (N)	80.5% (413)	73.8% (8,130)	χ2 (1) = 11.36;
Male, % (N)	19.5% (100)	26.2% (2,880)	*P* = 0.001
			
**Age in years, M (SD)**	53.06 (9.94)	-	-
			
**Years in the profession, M (SD)**	12.40 (9.90)	16.24 (10.64)	t(501) = −8.69; *P* < 0.001
**Cluster**			
Psychodynamic, % (N)	21.0% (106)	29.1% (2,794)	χ2 (3) = 53.62;
Humanistic, % (N)	47.6% (240)	32.3% (3,108)	*P* < 0.001
Systemic, % (N)	23.0% (116)	25.7% (2,473)	
Behavioral, % (N)	8.3% (42)	12.8% (1,233)	
**Region**			
Vienna	38.8% (199)	41.7% (4,197)	χ2 (8) = 29.44;
Upper Austria	12.3% (63)	10.3% (1,039)	*P* < 0.001
Lower Austria	18.9% (97)	12.8% (1,284)	
Carinthia	2.7% (14)	4.7% (474)	
Styria	7.2% (37)	9.6% (968)	
Tyrol	8.8% (45)	8.0% (807)	
Salzburg	7.8% (40)	7.6% (764)	
Burgenland	1.9% (10)	1.8% (184)	
Vorarlberg	1.6 % (8)	3.4% (342)	

Total numbers do not always sum up to *n* = 513 in participating psychotherapists and *n* = 11,156 in licensed psychotherapists, as not all sociodemographic data were available for all psychotherapists.

The majority (97.5%) of the psychotherapists worked in private practice. Only 2.5% were employed solely in institutions. Of those psychotherapists working in private practice, 15.6% additionally were employed in an outpatient facility and 5.4% in an inpatient facility. On average M (SD): 18.65 (9.02), median: 19.0 patients were treated per psychotherapist and week.

The matched sub-sample from the Austrian general population was surveyed simultaneously as the sample of psychotherapists and comprised *n* = 513 individuals. They were 52.33 ± 13.39 years old, and 80.5% were female. Age [*t*_(944.74)_ = −1.004; *P* = 0.32] and gender (χ2(1) = < 0.001; *P* = 1.0) did not differ between the psychotherapists and the general population sample.

### Mental health indicators in psychotherapists vs. the general population

Univariate analyses (Table 2) revealed higher mental wellbeing (*P* = 0.032) and less perceived stress in psychotherapists compared to the general population (*P* < 0.001). Also, mean values for depressive, anxiety and insomnia symptoms were lower in psychotherapists (*P* < 0.01).

**Table 2:** Mean scores for mental wellbeing, depressive symptoms, anxiety symptoms, insomnia symptoms and perceived stress levels in the general population (*n* = 513) and psychotherapists (*n* = 513).

**Variable**	**Group**				***P***	***Cohen‘s d***
	**General population (*****n*** = **513)**		**Psychotherapists (*****n*** = **513)**			
	**M**	**SD**	**M**	**SD**		
Wellbeing (WHO-5)	54.11	25.56	57.12	18.54	*t*_(934.02)_ = −2.15; *P* = 0.032	0.14 [0.01, 0.26]
Depressive symptoms (PHQ-2)	1.70	1.65	1.29	1.14	*t*_(908.83)_ = 4.60; *P* < 0.001	−0.29 [−0.41, −0.17]
Anxiety symptoms (GAD-2)	1.39	1.62	1.12	1.13	*t*_(913.07)_ = 3.15; *P* = 0.002	−0.19 [−0.32, −0.07]
Insomnia symptoms (ISI-2)	2.80	1.99	2.42	1.82	*t*_(1, 024)_ = 3.16; *P* = 0.002	−0.20 [−0.32, −0.08]
Stress level (PSS-10)	16.06	7.21	12.19	6.00	*t*_(991.27)_ = 9.35; *P* < 0.001	−0.58 [−0.71, −0.46]

*P, P*-values (2-tailed); M, mean score; SD, standard deviation, t, t-test; WHO-5, Wellbeing questionnaire of the World Health Organization (WHO); PHQ-2, Patient Health Questionnaire 2 scale; GAD-2, Generalized Anxiety Disorder 2 scale [assessing feeling nervous, anxious, or on edge (item 1) and not being able to stop or control for worrying (item 2)]; ISI-2, Insomnia Severity Index; PSS-10, Perceived Stress Scale 10.

The prevalences of clinically relevant depression, anxiety, insomnia and stress levels were lower in psychotherapists compared to the general population (*P* < 0.01; Table 3).

**Table 3:** Proportion of participants exceeding the cut-off scores for moderate depression/anxiety/insomnia and stress by group (*n* = 1,026).

**Variable**		**Group**		***P***
		**General population (*n* = 513)**	**Psychotherapists (*n* = 513)**	
Depression	% (*n*)	24.4% (125)	11.3% (58)	χ2 (1) = 29.86; *P* < 0.001
Anxiety	% (*n*)	17.9% (92)	10.9% (56)	χ2 (1) = 10.23; *P* = 0.001
Insomnia	% (*n*)	9.9% (51)	5.3% (27)	χ2 (1) = 7.99; *P* = 0.005
Moderate/high stress	% (*n*)	63.0% (323)	36.6% (188)	χ2 (1) = 71.05; *P* < 0.001

*P, P*-values (2-tailed); χ2, Chi-squared-test; Depression, ≥3 points on the Patient Health Questionnaire 2 scale; Anxiety, ≥3 points on the Generalized Anxiety Disorder 2 scale; Insomnia, ≥6 on the 2-item Insomnia Severity Index; Moderate/High Stress, ≥14 points on the Perceived Stress Scale 10.

**Figure 1:**
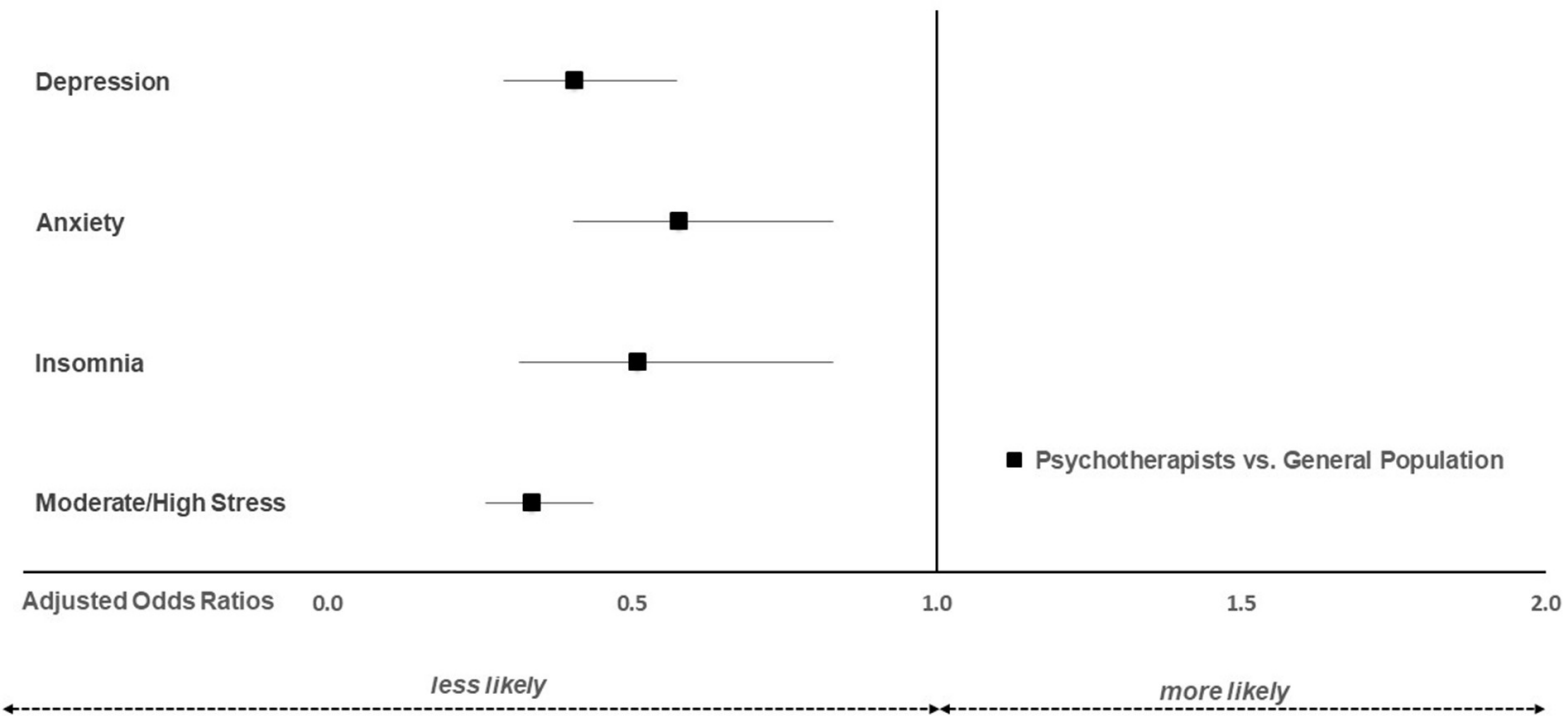
Adjusted odds ratios for clinically relevant depression, anxiety, insomnia and stress in psychotherapists (*n* = 513) vs. the general population (*n* = 513).

Multivariable analyses confirmed the univariate findings. As depicted in Figure 1, psychotherapists, compared to the general population, were less likely to experience clinically relevant depression (aOR 0.41; 95% CI: 0.29, 0.57), anxiety (aOR 0.58; 95% CI: 0.40, 0.83), insomnia (aOR 0.51; 95% CI: 0.31, 0.83) and moderate to high stress levels (aOR 0.34; 95% CI: 0.26, 0.44). Female gender increased the odds for depression (aOR: 1.63; 95% CI: 1.02, 2.61), anxiety (aOR: 1.90; 95% CI: 1.11, 3.25) and moderate/high stress (aOR: 1.44; 95% CI: 1.04, 1.99), whereas for insomnia no significant difference was observed (aOR: 1.87; 95% CI: 0.92, 3.84). With increasing age, the odds for depression (aOR: 0.97; 95% CI: 0.96, 0.98), anxiety (aOR: 0.98; 95% CI: 0.96, 0.99) and moderate/high stress (aOR: 0.98; 95% CI: 0.96, 0.99) decreased, whereas for insomnia no significant age effect was observed (aOR: 0.99; 95% CI: 0.97, 1.01).

## Discussion

This study shows that psychotherapists experience better mental health than the general population during the COVID-19 pandemic. Significantly lower odds for exceeding cut-offs for clinically relevant depression, anxiety, insomnia and stress (0.34–0.58) were observed for psychotherapists compared to the general population. Despite the better mental health status of psychotherapists relative to the general population, a significant proportion exceeded cut-offs for clinically relevant insomnia (5%), depression (11%), anxiety (11%) and stress (37%).

We assume that the reason for the better mental health of psychotherapists compared to the general population is due to several characteristics describing the group of psychotherapists, with high professional motivation, a secure social background and the possibility of independent time management being among the most critical factors.

Professional education for psychotherapists in Austria is subject to the Psychotherapy Act. Aspiring psychotherapists must be particularly motivated to practice their job since, in contrast to other legally recognized health professions, the training must be entirely funded by the candidates (48). It is extended over an average of 8.9 years (49) and somewhat selective through admission procedures to the specialized training. About 25% of the candidates drop out after the general training part (step one) without continuing the specialized training (step two). In addition, the curricula prescribe a high number of hours of self-experience or teaching therapy. This structure is supposed to select only candidates with higher reflective competence to remain in the process (48). Research has shown that improved reflective ability is associated with systematic attempts to develop it, a safe atmosphere, peer support and time to reflect (50). This is where the structural contexts of people's lives come into play, as they are the sources of security and social networks (51). Research on psychotherapy training in Austria assessed baseline and sociodemographic background data from a group of 197 psychotherapy trainees from Austria, finding that the group consisted mainly of individuals with satisfactory, financially secure life situations (52, 53). Also, other characteristics make the group distinct: 70% of the candidates have previous professional experience in the psychosocial field (49). At the same time, 73% of psychotherapists are academically trained (54). These background data do not necessarily suggest that all psychotherapists currently enjoy a high income. They may indicate that psychotherapists come from sociocultural milieus providing them with more than average knowledge and possibilities. Not only do socially secure and educated middle-class backgrounds protect the candidates from chronic high stress (51), but also education in itself is an essential social determinant of health (55), which contributes to explaining the relative resilience toward stress of psychotherapists compared to the general population.

Moreover, psychotherapists in Austria do not usually work full time, nor are most employed in health care institutions. According to a study conducted in 2019, the median number of hours of patient work in Austrian psychotherapists is 12 h (56). Another 12 h per psychotherapist have been reported to be spent on institutional psychotherapeutic activities and documentation activities (e.g., preparation of hourly protocols for case discussions). Adding all types of activities, the median weekly working time per psychotherapist in Austria before the pandemic was estimated to be 24 h (56). However, in the current survey, psychotherapists reported treating a median number of 19 patients per week, assuming the workload increased compared to pre-pandemic data. Corresponding to the data gathered in 2019, most participating psychotherapists worked in private practice, not in institutions. Compared to paid employees, research showed that self-employed individuals are more likely to be satisfied with their present jobs in terms of their work type (57) because their work provides more autonomy, flexibility, and skill utilization (58). These factors add to explaining why psychotherapists experience better mental health than the general population. In contrast, psychiatrists and other medical doctors seemed to be among the more burdened groups (14, 59). Whereas medical staff in hospitals often had to follow strict and changing protocols, experienced stress related to a high workload and did not feel valued (60), these challenges typical for institutions do not affect freelancers in the same way.

With the training each psychotherapist underwent during their professional education, we assume that they gained further resilience toward stress related to COVID-19 and other crises. Although opinions are divided on the issue of whether professionals with previous personal counseling are more effective in treating patients (61), research on the processes and outcomes of professionals' therapy has found respondents reporting improvement in behaviors, emotions or insights (62–64). A mindful approach to oneself acquired through self-experience and theoretical knowledge about mental hygiene may contribute to the psychotherapists' ability to cultivate resilience and self-care practices (65). In addition, fully trained psychotherapists in Austria are required by law to engage in supervision, which includes validating and processing clinically challenging situations and discussing areas such as work overload and “burnout avoidance strategies” (66). Taking time to acknowledge a patient's difficulties as a natural part of the therapeutic process can be an effective strategy to alleviate stress (21).

Better than average job security is likely to be a further factor preventing psychotherapists from stress in the previous 2 years. While work-related issues, including job security, were one of the three most frequently reported stressors in the general population after the first year of the pandemic (31), job security did not equally concern psychotherapists during the pandemic, as the demand for mental health services in the general population increased (30).

Differences in income might be another reason for better mental health among the psychotherapist group. Low pay is associated with increased risk for incident mental disorders (67) and is more likely in the general population than among psychotherapists. However, a German study found that considerable dissatisfaction with the financial situation was an outstanding stressor for psychotherapists (68). Low income as the main factor for the experience of better mental health among psychotherapists is also contradicted by the finding that the volunteers of the telephone counseling service (Telefonseelsorge) also experience better mental health than the general population (69) and that doctors who earn an above-average income have been shown to have worse mental health than the general population (70, 71).

Last but not least, it has to be highlighted that although psychotherapists experience better mental health than the general population, still a significant proportion exceeded cut-offs for clinically relevant disorders. Therefore, psychotherapists need to foster their self-care behaviors to manage their work-related distress to maintain mental health. Furthermore, psychotherapists who experience mental health problems themselves should seek professional support (e.g., supervision, personal therapy) to improve their quality of life as well as professional performance (16, 17).

This study has several limitations. First, no conclusion can be drawn whether the psychotherapists' mental wellbeing changed during the pandemic compared to the time before, as no pre-pandemic data on mental health in psychotherapists are available. Second, the response rate of licensed Austrian psychotherapists with an email address in 2022 was low. We suspect that a response rate around 8% in 2022, compared to around 25% who participated in a previous Austrian study in 2020 (22), is most likely due to the increased demand for psychotherapy throughout the pandemic. We assume that psychotherapists found less time to participate in a survey. The low response rate could also point to respondent bias, such as higher psychotherapists' participation with a higher preference for new technologies. Moreover, there is a slight possibility that the lower response rate in 2022 is related to worse mental health, meaning that those psychotherapists who experienced more stress skipped the survey in 2022. However, if this were the case, it probably would have also affected the general population. Third, all mental health indicators were based on self-reports and not confirmed by structured clinical interviews due to the online nature of the study. Fourth, although the samples were matched for age and gender, they were not matched for region, income, economic status, education level, marital status or being infected with SARS-CoV-2. In future studies education, economic status, marital status, and income need to be considered to elucidate whether the psychotherapists‘ training and personal qualities are associated with the better mental health status of the psychotherapists, or whether the differences are mainly due to differences in the socioeconomic status. However, as no associations of the region with all investigated outcome measures were observed, this variable was neither considered for matching the two datasets nor included in the final statistical analyses. Similarly, being infected with SARS-CoV-2 was assessed in the current survey but was not included in the paper as no association with mental health indicators was observed.

## Conclusion

Specific characteristics of the group of psychotherapists, such as high professional motivation, a secure social background and the possibility of independent time management, seem to contribute to more resilience of this sample compared to the general population. Further studies are required to elucidate the protective factors underlying these findings.

## Data availability statement

The raw data supporting the conclusions of this article will be made available by the authors, without undue reservation.

## Ethics statement

The studies involving human participants were reviewed and approved by Ethics Committee of the University for Continuing Education Krems, Austria (Ethical numbers: EK GZ 26/2018-2021 and EK GZ 11/2021-2024). The patients/participants provided their written informed consent to participate in this study.

## Author contributions

EH, YS, TP, CP, and AJ: conceptualization and methodology. SK and EH: formal analysis. EH: investigation and data curation. YS and EH: writing—original draft preparation. AJ, TP, SK, and CP: writing—review and editing. All authors have read and agreed to the published version of the manuscript.

## Conflict of interest

The authors declare that the research was conducted in the absence of any commercial or financial relationships that could be construed as a potential conflict of interest.

## Publisher's note

All claims expressed in this article are solely those of the authors and do not necessarily represent those of their affiliated organizations, or those of the publisher, the editors and the reviewers. Any product that may be evaluated in this article, or claim that may be made by its manufacturer, is not guaranteed or endorsed by the publisher.
